# Scaling-up essential neuropsychiatric services in Ethiopia: a cost-effectiveness analysis

**DOI:** 10.1093/heapol/czv093

**Published:** 2015-10-21

**Authors:** Kirsten Bjerkreim Strand, Dan Chisholm, Abebaw Fekadu, Kjell Arne Johansson

**Affiliations:** ^1^Department of Global Public Health and Primary Care University of Bergen Postbox 7804, N- 5020 Bergen,; ^2^World Health Organization, Geneva, Switzerland,; ^3^College of Health Sciences, School of Medicine, Department of Psychiatry, University of Addis Abeba, Addis Ababa, Ethiopia and; ^4^Institute of Psychiatry, Department of Psychological Medicine, King's College London, London, UK

**Keywords:** Cost-effectiveness, ethics, mental health, neuropsychiatric disorders

## Abstract

**Introduction **There is an immense need for scaling-up neuropsychiatric care in low-income countries. Contextualized cost-effectiveness analyses (CEAs) provide relevant information for local policies. The aim of this study is to perform a contextualized CEA of neuropsychiatric interventions in Ethiopia and to illustrate expected population health and budget impacts across neuropsychiatric disorders.

**Methods **A mathematical population model (PopMod) was used to estimate intervention costs and effectiveness. Existing variables from a previous WHO-CHOICE regional CEA model were substantially revised. Treatments for depression, schizophrenia, bipolar disorder and epilepsy were analysed. The best available local data on epidemiology, intervention efficacy, current and target coverage, resource prices and salaries were used. Data were obtained from expert opinion, local hospital information systems, the Ministry of Health and literature reviews.

**Results **Treatment of epilepsy with a first generation antiepileptic drug is the most cost-effective treatment (US$ 321 per DALY adverted). Treatments for depression have mid-range values compared with other interventions (US$ 457–1026 per DALY adverted). Treatments for schizophrenia and bipolar disorders are least cost-effective (US$ 1168–3739 per DALY adverted).

**Conclusion **This analysis gives the Ethiopian government a comprehensive overview of the expected costs, effectiveness and cost-effectiveness of introducing basic neuropsychiatric interventions.

Key Message

- A cost-effectiveness analysis of basic neuropsychiatric interventions in an Ethiopian setting based on a proposed set of interventions from the Ethiopian Ministry of Health. Depending on the willingness-to-pay several interventions can be viewed as cost-effective. A discussion on how to weight cost-effectiveness compared with other fairness concerns.

## Introduction

Mental and behavioral disorders contribute to around 19% of all years of life lost due to disability (YLD) in Eastern Sub-Saharan Africa (Vos *et al*. 2012). Even though effective treatment exists, major depression is the second leading cause of YLD both globally and in the sub-region of Eastern Sub-Saharan Africa. Mental illness greatly influences the patients’ overall health, economic situation and social integration. The prevalence and severity of other conditions, such as cardiovascular diseases, communicable diseases and intentional/unintentional injuries, are associated with poor mental health (Prince *et al*. 2007). In addition, mental disorders are a financial risk factor and can substantially influence ability to work and household prosperity. Poor populations are also at higher risk for mental illnesses (Lund *et al*. 2010). The mentally ill are a vulnerable and often stigmatized group. Ethiopian studies have shown that mental illness is associated with a high degree of stigma (Shibre *et al*. 2001; Assefa *et al*. 2012).

Scaling up mental health services in Ethiopia is critical due to the shortage of neuropsychiatric treatment and the massive lack of trained personnel in all mental health professions, as in most low-income countries (Saxena *et al*. 2007; mhGAP-Ethiopia Working Group 2010; Bruckner *et al*. 2011; Federal Democratic Republic of Ethiopia Ministry of Health 2012). This is recognized in the current National Mental Health Strategy, which has an ambitious goal of addressing the mental health needs of the entire Ethiopian population (Federal Democratic Republic of Ethiopia Ministry of Health 2012). The National Mental Health Strategy explicitly states that efficiency and cost-effectiveness are important in the scale-up of mental health interventions. The basic scale-up scenario in the National Mental Health Strategy targets treatment for depression, psychosis, bipolar disorder and epilepsy, which are key interventions in the WHO mental health Gap Action Programme (mh-GAP) (mhGAP-Ethiopia Working Group 2010). The strategy identifies low budgets as a weakness and indicates that these are a threat to implementing mental health services (Federal Democratic Republic of Ethiopia Ministry of Health 2012). Evidence on the cost-effectiveness of the basic scale-up scenario is therefore important. However, the cost-effectiveness of these interventions in the Ethiopian context has not been evaluated. Evidence from the latest regional cost-effectiveness analysis (CEA) on neuropsychiatric disorders (Chisholm and Saxena 2012) is not necessarily sensitive to local variations; there are significant variations in results between regional and national CEAs (Reinap *et al*. 2005; World Health Organization 2006; Gureje *et al*. 2007; Ayuso-Mateos *et al*. 2008; Chisholm *et al*. 2008; Salomon *et al*. 2012a). A contextualized CEA can provide important information for prioritizing decisions as the mental health policy is being scaled up in Ethiopia. The aim of this study is: (1) to do a contextualized CEA for an Ethiopian setting examining a range of interventions for depression, schizophrenia, bipolar disorder and epilepsy; and (2) to illustrate the differences in expected population health and budget impact across these disorders.

## Materials and methods

### Study design and population

This is a generalized CEA, which has been performed in an Ethiopian setting (Box 1) from a health provider perspective. All costs and health benefits were discounted at 3% and no age weights were applied. Assumptions and variables in existing CEA models from WHO-CHOICE on neuropsychiatric disorders were revised. The best available local data on epidemiology, intervention efficacy, current coverage, item prices, salaries and quantity assumptions replaced the existing assumptions in the model (see Box 1, Tables 2 and 3 and Appendix). Specific data from Ethiopia have been used when available; however, the availability of data is scarce and we have therefore supplemented it with regional data and estimates based on the calculation framework from WHO-CHOICE.

Box 1.Overview of key characteristics of Ethiopia

**Table:** 

	Characteristics		Year	Source
Demography	Total national population	94,100,756	2013	World Bank Data
	Proportion below poverty line of Int$1.25	30.7%	2011	World Bank Data
	Proportion below poverty line of Int$2	66.0%	2011	World Bank Data
	Expenditure on health (USD/capita)	17.6	2012	World Bank Data
Human resources	Psychiatrists^*^	40	2010	MoH Ethiopia
	Psychiatric nurses	461	2010	MoH Ethiopia
	Psychologists^**^	14	2010	MoH Ethiopia
	Social Workers	3	2010	MoH Ethiopia
	Occupational therapists	0	2010	MoH Ethiopia
	Psychiatric beds	326	2010	mhGAP-Ethiopian Working Group
Epidemiology	Prevalence depression	2.2%	2010	Fekadu *et al*. (2007)
	Prevalence schizophrenia	0.5%	2010	mhGAP-Ethiopian Working Group
	Prevalence bipolar disorder	0.5%	2010	mhGAP-Ethiopian Working Group
	Prevalence epilepsy	1%	2010	mhGAP-Ethiopian Working Group
	Prevalence alcohol drinking problem	2.2–3.7%	2010	mhGAP-Ethiopian Working Group
Current coverage	Depression	<1%	2010	mhGAP-Ethiopian Working Group
	Schizophrenia	<1%	2010	mhGAP-Ethiopian Working Group
	Bipolar Disorder	1%	2010	mhGAP-Ethiopian Working Group
	Epilepsy	5%	2010	mhGAP-Ethiopian Working Group

^*^30 of the 40 psychiatrists are located in Addis Abeba.

^**^none with training as clinical psychologists.

**Figure 1.**
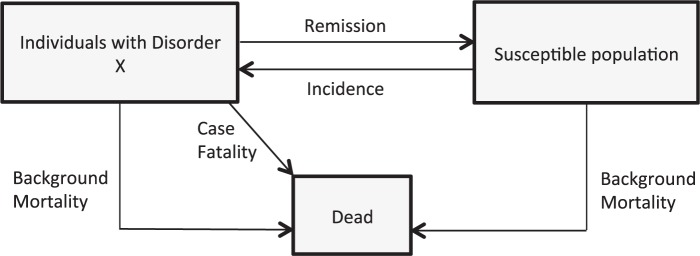
Model explanation.

The software used in the analysis was population model (PopMod), which is a population-based multi-state analytical health economic tool developed by WHO-CHOICE. A detailed description of the method can be found elsewhere (Edejer *et al*. 2012). The model is based on three health states: condition X, susceptible without condition X and death. In the first year, the total Ethiopian population (exact age groups) is distributed into the three health states. Jumps between health states are determined by disease-specific incidence rates, remission rates, case fatality rates and age-specific mortality rates for susceptible groups (Figure 1).

Each model-run is one year and the model runs for 100 years, but interventions are only applied for the first 10-year period. Health benefits of the programme are assessed over a 100-year period in order to include the long-term benefits of prevention or treatment late in life.

The newest age-specific demographic distribution in Ethiopia from UN populations 2010 was used (UN Population Devision). Epidemiological data on prevalence, incidence, case-fatality, health state valuation, disability weights, birth rates and mortality have been collected from the latest available update of the Global Burden of Disease (GBD-2010) study (Salomon *et al*. 2012b). Where country-specific data on epidemiological factors was available, this has been used; where this has not been available, we have used data from the East African sub-region. The epidemiological input was compared with and validated for Ethiopian epidemiological studies (Tekle-Haimanot *et al*. 1997; Awas *et al*. 1999; Kebede and Alem 1999a,b; Kebede *et al*. 1999, 2003, 2006; Fekadu *et al*. 2007; Negash *et al*. 2005; Almu *et al*. 2006). The GBD data have been supplemented with information collected from experts at Amanuel Psychiatric Hospital and the Ministry of Health in Ethiopia, and from a literature review. The mh-GAP implementation project estimated the coverage level of the different mental health interventions in Ethiopia in 2010; these estimates were used as the current coverage levels in the model (mhGAP-Ethiopia Working Group 2010).

### Selection of disorders and interventions

A complete model of care was used, and the scale-up scenarios are close to what is described in the National Mental Health Strategy in Ethiopia and the WHO-supported Ethiopia mh-GAP programme. Treatment is provided primarily by generalists in a primary-care setting (mhGAP-Ethiopia Working Group 2010; Federal Democratic Republic of Ethiopia Ministry of Health 2012). An overview of the interventions included in the analysis is presented in Table 1. The interventions in this analysis have been analysed in the regional CEA from 2012 (Chisholm and Saxena 2012); however, the interventions included in this analysis target disorders in the basic scenario of the National Mental Health Strategy. The analysis was not limited to interventions in the basic scenario of the National Mental Health Strategy. Analyses of newer medications for bipolar disorder and schizophrenia and psychotherapy for depression were added, since these are considered in treatment guidelines from higher-income settings like the UK (National Institute of Health and Clinical Excellence 2006; National Institute of Health and Clinical Excellence 2009a,b). The National Mental Health Strategy suggests treating bipolar disorders using mainly antipsychotics. This option was not included in the analysis due to the very limited evidence on long-term treatment effects (Geddes and Miklowitz 2013). The target coverage varies between the disease categories and reflects the targets set in the basic scale-up scenario (Federal Democratic Republic of Ethiopia Ministry of Health 2012). We adhere to the ICD-10 definitions of disorders.

**Table 1.** Overview of interventions

Disorder	Intervention number	Intervention description	Coverage
**Depression**	DEP1	Older antidepressants (TCA)	30%
	DEP2	Newer antidepressants (SSRI)	30%
	DEP3	Psychotherapy	30%
	DEP4	Older antidepressants (TCA) and psychotherapy	30%
	DEP5	Newer antidepressants (SSRI) and psychotherapy	30%
	DEP6	Maintenance: Older antidepressants (TCA) and psychotherapy	30%
	DEP7	Maintenance: Newer antidepressants (SSRI) and psychotherapy	30%
**Schizophrenia**	SCZ1	Typical antipsychotics	75%
	SCZ2	Atypical antipsychotics	75%
	SCZ3	Typical antipsychotics + psychosocial treatment	75%
	SCZ4	Atypical antipsychotics + psychosocial treatment	75%
	SCZ5	Case id + manager: Typical antipsychotics and psychosocial treatment	75%
	SCZ6	Case id + manager: Atypical antipsychotics and psychosocial treatment	75%
**Bipolar disorder**	BIP1	Older mood stabilizer (Lithium)	50%
	BIP2	Newer mood stabilizer (Valproate)	50%
	BIP3	Older mood stabilizer (Lithium) and psychosocial treatment	50%
	BIP4	Newer mood stabilizer (Valproate) and psychosocial treatment	50%
**Epilepsy**	EPI1	Older antiepileptic treatment (Phenobarbital)	75%
	EPI2	Newer antiepileptic treatment (Carbomazepine)	75%

### 2.3 Estimation of intervention effectiveness

Health benefits are measured in disability adjusted life years (DALYs). A null scenario is estimated first, where the health state of the Ethiopian population is calculated if no interventions were available (the model ‘rewinds’ the demography by downscaling the current coverage of interventions to zero). The null scenario serves as a baseline for assessing the incremental effects of increasing intervention coverage. When compared with the null scenario, the effect of treatments is incremental reductions in disability weight values, fatality rates and incidence of mental illness or increasing remission rates (see Table 2 and Appendix). In addition, effects are adjusted by treatment coverage and adherence to treatment. Estimates of the efficacy of interventions have been updated with results from systematic reviews, meta-analyses and Randomized Controlled Trials (Table 2). Evidence from Ethiopia was considered most valuable, but such evidence was not available.

**Table 2.** Effectiveness assumptions

Treatment	Outcome	Efficacy	Drug/other treatment	Source
**Depression**				
Treatment with older Antidepressants (TCA)	Remission	29%	Amtriptyline (100 mg daily)	Arroll *et al*. (2005); Andrews *et al*. (2000)
	Disability	−31%		
Treatment with newer Antidepressants (SSRI)	Remission	28%	Fluoxetine (20 mg daily)	Arroll *et al*. (2005); Andrews *et al*. (2000)
	Disability	−31%		
Psychotherapy	Remission	28%	Cognitive therapy (16–20 h)	de Maat *et al*. (2007); Andrews *et al*. (2000)
	Disability	−31%		
Comibnation of AD and psychotherapy	Remission	38%	Amtriptyline/Fluoxetine, cognitive therapy	de Maat *et al*. (2007); Andrews *et al*. (2000)
	Disability	−31%		
Proactive care	Remission	38%	Amtriptyline/Fluoxetine, cognitive therapy	de Maat *et al*. (2007); Andrews *et al*. (2000); Geddes *et al*. (2003)
	Disability	−31%		
	Incidence	−35%		
**Schizophrenia**				
Older antipsychotics	Disability	−13%	Chlorpromazine 300 mg daily/Fluphenazine deconate 25 mg daily	Leutch *et al*. (1999)
Newer antipsychotics	Disability	−14%	Risperidone 4 mg daily	Leutch *et al*. (1999)
Older antipsychotics + psychosocial treatment	Disability	−23%	Chlorpromazine 100 mg daily/Fluphenazine deconate 25 mg daily, supportive psychosocial treatment	Mojtabai *et al*. (1998); Leutch *et al*. (1999)
Newer antipsychotics + psychosocial treatment	Disability	−24%	Risperidone 4 mg daily, supportive psychosocial treatment	Mojtabai *et al*. (1998); Leutch *et al*. (1999)
Case-management with older antipsychotics	Disability	−24%	Chlorpromazine 300 mg daily/Fluphenazine deconate 25 mg daily	Mojtabai *et al*. (1998); Leutch *et al*. (1999)
Case-management with newer antipsychotics	Disability	−25%	Risperidone 4 mg daily	Mojtabai *et al*. (1998); Leutch *et al*. (1999)
**Bipolar disorder**				
Older mood stabilizer	Disability	−70%	Lithium Carbonate	Goodwin *et al.* (1990); Angst *et al*. (2000); Bowden *et al*. (2000); Tondo *et al*. (2001)
	Case-fatality	−65%		
Newer mood stabilizer	Disability	−65%	Sodium Valproate	Goodwin *et al*. (1990); Bowden *et al* 1994; Angst *et al*. (2000); Bowden *et al*. (2000); Tondo *et al*. (2001)
	Case-fatality	−65%		
Older Mood Stabilizer + psychosocial	Disability	−70%	Lithium Carbonate, supportive psychososial treatment	Goodwin *et al*. (1990); Angst et al. (2000); Bowden *et al*. (2000); Tondo *et al*. (2001); Lam *et al.* (2009)
	Case-fatality	−65%		
Newer mood stabilizer + psychosocial	Disability	−65%	Sodium Valproate, supportive psychosocial treatment	Goodwin *et al.* (1990); Bowden *et al*. (1994, 2000); Angst *et al*. (2000); Tondo *et al*. (2001); Lam *et al* (2009)
	Case-fatality	−65%		
**Epilepsy**				
Older antiepileptic	Remission	60%	Phenobarbital 100 mg daily	Annegers *et al*. (1979); Goodridge, *et al*. (1983); Placenica *et al*. (1994); Cockerell *et al*. (1995) Tudur Smith *et al*. (2009)
	Disability	−43%		
Newer antiepileptic	Remission	60%	Carbamazepine 600 mg daily	Annegers *et al*. (1979); Goodridge *et al*. (1983); Placenica *et al*. (1994); Cockerell *et al*. (1995) Tudur Smith *et al*. (2009)
	Disability	−43 %		

### Estimation of intervention costs

Unit prices and quantities needed at different levels of the health system (e.g. drugs, salaries, hospital bed-day costs etc.) are used to calculate costs. Facility costs (or direct patient costs), programme costs and training costs are included in total costs (see appendix). The patient out-of-pocket expenditures related to help-seeking and the productivity impact for the patient, household and families are not included in the analysis. Finally, total 10-year costs are combined with the respective effectiveness of the interventions.

Amanuel Psychiatric Hospital was the main source for local unit prices of laboratory tests and salaries (see Table 3). This information was cross-verified with data from Black Lion Hospital and the Ministry of Health in Ethiopia. Amanuel Psychiatric Hospital is a specialist psychiatry hospital and Black Lion Hospital is a general teaching hospital in Addis Ababa. Ethical clearance from the Institutional Review Board at the Medical Faculty of Addis Ababa University was obtained for collection of costs data at the institutions. Unit prices were coherent across all of these governmental institutions. Default unit cost estimates for drugs were taken from the International Drug Price Indicator Guide (http://erc.msh.org), where the lowest ‘supplier price’ could be identified. The costs for drugs from this source were compared with Amanuel Psychiatric Hospital’s prices for drugs for patients. When Amanuel Psychiatric Hospital provided a lower price for a drug, this price was used. Average unit costs of bed-days and outpatient visits at primary and secondary health care levels were estimated using the WHO-CHOICE econometric analysis (Adam *et al*. 2003; Adam and Evans 2006). Unit costs for bed-days and outpatient visits include all cost components except drugs and laboratory tests. See Appendix for more details on cost and quantity assumption.

**Table 3.** Cost assumptions

**Salaries**		(Source: Amanuel Psychiatric Hospital, Black Lion Hospital, Ministry of Health Ethiopia)
	**Yearly salary (ETB)**	
Medical director	91 428	
Psychiatric specialist	83 988–91 428	
General practitionar	40 968	
Nurse	19 998–35 868	
Public health worker	27 000–35 868	
Health worker	17 124	
Occupational therapists	31 200	
Psychologist	29 112	
Social worker	31 224	
Pharmacist	18 426–40 968	
Labratory technichian	30 168	
Secretary	19 578	

**Lab tests**		(Source: Amanuel Psychiatric Hospital, Black Lion Hospital)

	**Unit Price ETB (per test)**	
Complete blood count package	45	WBC, RBC, Hb, hct, Plt and three differnitals
Hb	10	
Iron	20	
Kidney function test	15	Creatinine
Electolytes	75	Includes K+, Na+, Cl-, Ca+2
Liver function tests	75	Bil-tot, bil-dir, ALAT, ASAT, ALP
Thyroid tests	180	Includes TSH and FT4
Glucose	15	
Lithium level	51	

**Drugs**		(Source: http://erc.msh.org Nov 2013)
	**Unit price US$ (per tabl/amp)**	

Amtriptyline 25 mg tablet	0.0036	
Fluoxtine 20 mg capsule	0.0353	
Haldolperidol 5 mg tablet	0.0140	
Fluphenazine Decanoate 25 mg/1 ml injection	0.5958	
Risperidone 2 mg tablet	0.0085	
Triexiphenidyl Hcl 5 mg tablet	0.0063	
Lithium Carbonate 300 mg tablet	0.8000	Amanuel Psychiatric Hospital
Sodium Valproate 200 mg tablet	0.0315	
Phenobarbital 100 mg tablet	0.0041	
Carbamazapine 200 mg tablet	0.0148	
**Insitutional costs, training costs and Programme costs**		(Based on WHO-CHOICE framework)
GDP per capita (Int$)	1,354	World Bank 2013
GDP per capita (US$)	498	World Bank 2013
PPP conversion factor (ETB per Int$)	6.9	World Bank 2013
Offical exchange rate (ETB per US$)	17.7	World Bank 2012
% of population that are urban	17.5 %	World Bank 2013

Programme costs not directly linked to the providing health facility were analysed separately for each intervention. These costs include planning and administration, training of staff and monitoring and evaluation at a national, provincial and district level. Programme quantities are based on WHO-CHOICE expert estimates.

### Estimation of cost-effectiveness

Cost-effectiveness was assessed by calculating incremental cost-effectiveness ratios (ICERs). The ICER was calculated in comparison to the null scenario. The current situation has been excluded from the analysis, as it is close to the modelled null scenarios: the Ministry of Health in Ethiopia has estimated that the current coverage of many basic interventions is only about 1% (mhGAP-Ethiopia Working Group 2010). WHO recommends using a willingness-to-pay threshold defined by the nation’s GDP. Where interventions falling under one GDP per capita are considered highly cost-effective and interventions with an ICER below three GDP per capita are considered cost-effective (Commision on Macroeconomics and Health 2001). However, Revill *et al*. (2014) states ‘the use of WHO-recommended cost-effectiveness benchmarks of one and three times GDP per capita lacks a theoretical or empirical basis’ (Revill *et al*. 2014). We will present the results according to three possible willingness-to-pay thresholds: (1) US$ 100; (2) one times GDP—US$ 500; (3) three times GDP—US$ 1500.

### Uncertainty analysis

To perform the uncertainty analysis, we used the WHO-CHOICE software programme Monte Carlo (MC) League (Hutubessy *et al*. 2001; Baltussen *et al*. 2002). The MC League represents uncertainty regarding costs and effects in the form of stochastic league tables and the analysis allows for covariance between the costs and effectiveness. We conducted a probabilistic MC uncertainty analysis of the results with 1000 simulation runs and a truncated normal distribution to assess the certainty of the results. We used baseline results together with variation coefficients ranging between 15 and 25%. The results of the analysis are presented as willingness-to-pay curves and scatter plots of the uncertainty range for both costs and effects.

## Results

**Figure 2.**
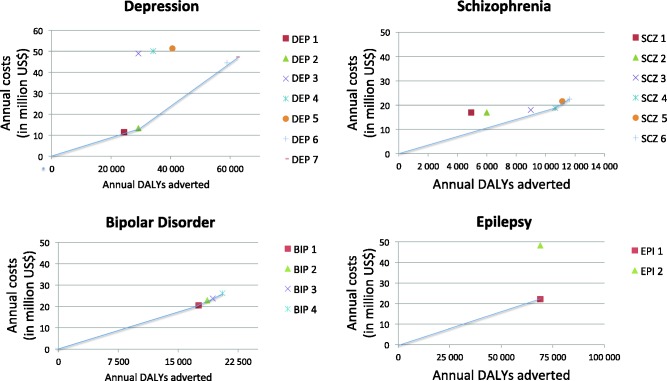
Expansion path of interventions.

Table 4 provides an overview of the costs, effectiveness and cost-effectivenessratios for the interventions suggested in the National Mental Health Strategy. Figure 2 shows expansion paths for all disorders, suggesting how to scale up the interventions according to variations in societal willingness-to-pay for mental health care.

**Table 4.** Cost-effectiveness overview

Disease Category	Intervention number	Annual costs (in million US$)	Annual DALYs adverted^a^	ACER	ICER
Depression	DEP1	11.44	24 340	470	D^b^
	DEP2	13.31	29 136	457	457
	DEP3	48.90	29 136	1678	D
	DEP4	49.97	34 075	1467	D
	DEP5	51.24	40 576	1263	D
	DEP6	44.55	58 926	756	D
	DEP7	47.21	62 193	759	1026
Schizophrenia	SCZ1	16.96	4925	3444	D
	SCZ2	16.96	5992	2831	D
	SCZ3	17.98	8988	2001	D
	SCZ4	18.84	10 650	1769	1769
	SCZ5	21.60	11 126	1941	D
	SCZ6	22.46	11 617	1933	3739
Bipolar disorder	BIP1	20.50	17 552	1168	1168
	BIP2	22.90	18 636	1229	D
	BIP3	23.71	19 333	1227	1807
	BIP4	26.14	20 530	1273	2023
Epilepsy	EPI1	22.16	68 935	321	321
	EPI2	48.20	68 935	699	D

ACER, average cost-effectiveness ratio (US$ per DALY adverted); ICER, incremental cost-effectiveness ratio (US$ per DALYs adverted)

^a^Discounted, not age-weigthed.

^b^D, dominated (intervention is more costly and/or less effective than other more efficient interventions).

We can consider the results depending on three willingness-to-pay thresholds: (1) US$ 100; (2) US$ 500 (one GDP); (3) US$ 1500 (three GDP). If the willingness-to-pay threshold is set to US$ 100 none of the interventions targeting neuropsychiatric disorders would be implemented. Implementing alternative 2, a threshold of one GDP (US$ 500), a basic scenario package would include treatment of epilepsy with older anti-epileptic (US$ 321 per DALY adverted) and newer antidepressants and psychotherapy, with maintenance treatment for depression (US$ 457 per DALY adverted). Treatments for schizophrenia and bipolar disorder would be excluded. A scale-up of these interventions will cost US$ 35.5 million annually. The new health budget would then be US$ 18.0 per capita, a 2.1% increase compared with the 2012 health budget (World Bank). The expected health gain this scale up is an additional 98 000 healthy life years.

Implementing Alternative 3, threshold of three GDP (US$1500), a basic scenario package would include treatment of epilepsy with older anti-epileptic (US$ 321 per DALY adverted), treatment for depression with newer antidepressants and psychotherapy with maintenance treatment (US$ 1026 per DALY adverted) and treatment with Lithium and psychosocial treatment for bipolar disorder (US$ 1168 per DALY adverted). The most cost-effective intervention for schizophrenia—risperidone with psychosocial supportive therapy (US$ 1769 per DALY adverted)—would be excluded. A scale-up of these will cost US$ 89.9 million annually. The new health budget would then be US$ 18.6 per capita, a 5.4% increase compared with the 2012 health budget (World Bank). The expected health gain this scale up is an additional 149 000 healthy life years.

**Figure 3.**
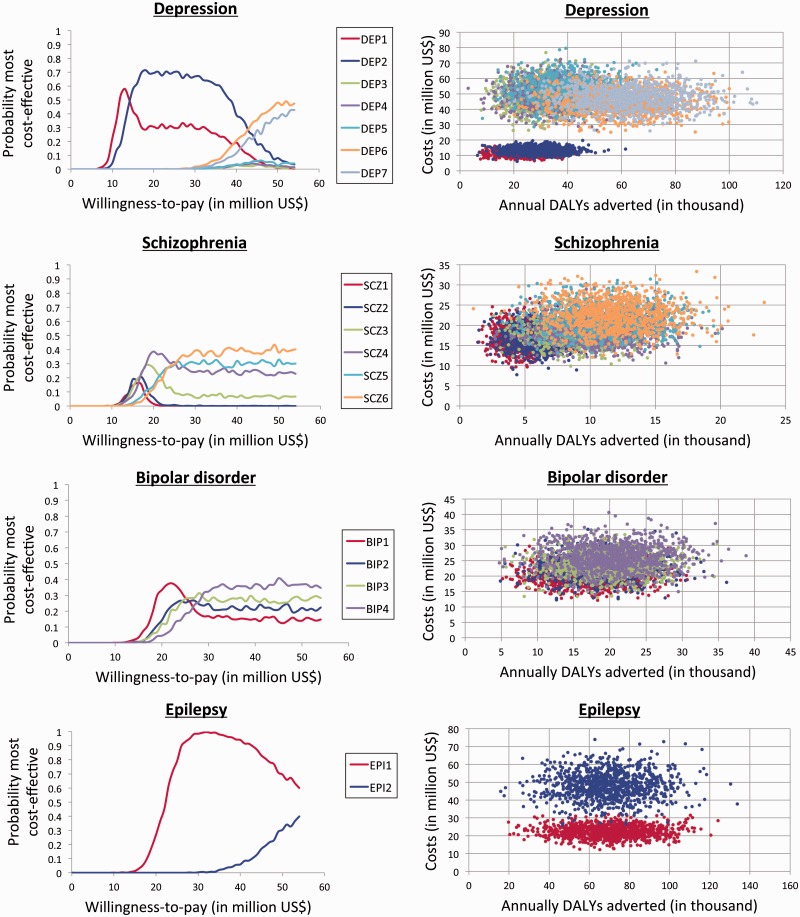
Sensitivity analysis.

The size of the health budget, or willingness to pay for a treatment, has substantial impact on the certainty of the results (see Figure 3). For depression, the probability curves for intervention DEP1 and DEP2 are roughly within the same range and it is therefore difficult to conclude whether newer or older antidepressants are most cost-effective. Due to lack of evidence, the uncertainty of the results is noteworthy, especially for interventions targeting schizophrenia and bipolar disorder.

## Discussion

This analysis assesses the cost-effectiveness of different eligible interventions targeting depression, schizophrenia, bipolar disorder and epilepsy. The three presented scale-up scenarios assume three different willingness-to-pay thresholds. Applying the lowest threshold of US$ 100 for a year of healthy life (Alternative 1) would result in there being no mental health care interventions selected for scale-up in Ethiopia (since all studied strategies exceed this level of efficiency). By including cost-effective neuropsychiatric intervention strategies under either Alternative 2 or 3, the total health budget, would increase by 2.1 and 5.4%, respectively. Even at the highest threshold used in Alternative 3 (US$ 1500 per health life year), treatment for schizophrenia would be excluded if a strict health-maximizing approach was pursued since even the most cost-effective strategy—treatment with newer antipsychotic medication (generically produced risperidone) plus psychosocial therapy—has a cost-effectiveness ratio above this fixed point.

Although it is evidently important to establish efficient pathways towards universal coverage, concerns regarding fairness also need to be taken into account in the formation of public health priorities (Norheim *et al*. 2013). This is often seen in actual policy decision-making, where policies often deviate from crude cost-effectiveness rankings (Dixon and Welch 1991). In particular, financial protection and disease severity are relevant fairness concerns that are not taken into account by crude ICER rankings (Norheim *et al*. 2013). Severity of disease was considered the leading priority in a survey conducted among key stakeholders in Uganda (Kapiriri *et al*. 2004; Kapiriri and Norheim 2004) and is a principle that has been theoretically well argued for (Nord 1993; Ottersen 2013). Schizophrenia, e.g. is highly disabling, occurs at a young age and pushes households into poverty; it can therefore be considered as a more severe condition than others under consideration (Daniels and Sabin 2002). If we introduce severity as an additional criterion, it will likely shift the ranking for treatment of schizophrenia. As of today, there are few methods available for explicit handling of such trade-offs between health maximization and concerns such as severity of disease and financial protection in economic evaluations regarding health care. Thus, there is a need to develop methods to incorporate cost-effectiveness with other relevant criteria in a priority-setting analytical framework.

In the search for efficiency, it is also crucial not to jeopardize the quality of care or service user outcomes. In the care model used in this analysis, we scale up mental health care in a primary care setting with referral as needed to specialized inpatient care. For conditions like schizophrenia, inpatient care remains a key cost driver (as can be seen in Supplementary Table S2 of the appendix). The National Mental Health strategy in Ethiopia suggests task shifting as an important strategy to reduce personnel costs (Federal Democratic Republic of Ethiopia Ministry of Health 2012). They suggest low- and middle-level health workers can undertake certain responsibilities carried out to date by specialists and primary care doctors. However, there is currently lack of evidence on how such task shifting could impact the quality of care. There are now studies being conducted in rural Ethiopia on task shifting of schizophrenia care (Lund *et al*. 2015). If quality of care and health outcomes can be maintained through such an approach, the cost-effectiveness of schizophrenia care may be substantially reduced.

We have chosen to use PopMod for this analysis, as this model has been used for similar studies in other low-income countries. It allows us to compare the results to previous studies as well as other disease categories. However, the method has limitations. It is a mathematical model and, therefore, is not as context-specific as an empirically based trial. The parameter assumptions are drawn from international as well as national data. The major limitation of this study is the limited availability of local data. There are few primary studies on specific costs or the efficacy of neuropsychiatric treatments in the Ethiopian healthcare system. Further, the costs data gathered in Ethiopia are mainly from Amanuel Psychiatric Hospital, Black Lion Hospital and the Ethiopian Ministry of Health. The hospitals are both located in Addis Ababa, while 81% of the population lives in rural areas. At the time of data collection, Amanuel Psychiatric Hospital was the only psychiatric hospital in the country. We have therefore relied on these three sources and estimates in the WHO-CHOICE model to calculate costs. The interventions in the analysis are focused on community-based treatment. In the model, we have estimated that there will be extra logistic expenditures due to transportation of, e.g. drugs. The salaries will not differ, as they are based on the Ethiopian Ministry of Health national tariffs.

The analysis takes a health provider perspective; costs do not include out-of-pocket costs related to help-seeking or loss of income due to loss in productivity. The health expenditures of patients need to be assessed, as it is clear that neuropsychiatric disorders have a large impact on all aspects of life, both for the patient and his/her family. The impact of the interventions on the patients’ ability to work, caretakers’ responsibility for the patient as well as the ability to have a job and costs in seeking help from both traditional and western health care providers are aspects that are not studied in this analysis. As an addition to this analysis, an extended CEA could be performed, such that the expected non-health benefits and financial protection for the respective interventions can be evaluated (Verguet *et al*. 2014).

Direct comparisons of many of the results in this contextualization and the regional CEA on mental health care (Chisholm and Saxena 2012) are complicated by the extensive revisions of local input assumptions as well as by differences in reporting year and currency, changing drug processes and age-weighting procedures. However, by comparing the results of this analysis to an earlier regional analysis that also removed age weights and reported in US$ (Hyman *et al*. 2006), it becomes apparent that many of the interventions studied at the national level have a lower budget impact than the regional study. This is in line with other WHO-CHOICE contextualization studies in Chile, Estonia, Mexico, Nigeria, Spain, Sri Lanka and Thailand (Reinap *et al*. 2005; World Health Organization 2006; Gureje *et al*. 2007; Ayuso-Mateos *et al*. 2008; Chisholm *et al*. 2008; Salomon *et al*. 2012a), which have likewise indicated that regional studies are not always that sensitive to local variations in epidemiology, costs and coverage. However, the major trends are similar between regional, global and contextualized CEAs; e.g. all of these analyses estimate that interventions for schizophrenia are the least cost effective. In addition, it is important to adjust for fluctuations in drug prices, as they are constantly changing and thus influencing the costs and cost-effectiveness of the interventions. Drug prices are often major contributors to the total costs of interventions in low- and middle-income countries. This was seen in a CEA in Nigeria where the prices of atypical antipsychotics were very high at the time of the analysis in Nigeria, and this drove the cost-effectiveness rates higher compared with the regional CEA (Gureje *et al*. 2007).

## Conclusions

Neuropsychiatric conditions contribute to a major burden of disease in Ethiopia, and the current coverage of neuropsychiatric interventions is low in Ethiopia. Our analysis provides an overview of the expected costs, effectiveness and cost-effectiveness ratios for different possible interventions targeting depression, schizophrenia, bipolar disorder and epilepsy. The limit the Ethiopian governments set for willingness-to-pay will determine which neuropsychiatric interventions could be provided for the Ethiopian people under a health maximization approach. Decisions on how to best scale up the Ethiopian health system should be made comparing interventions across disorders and medical disciplines. Ultimately, the decision should be based on both CEAs and an ethical discussion on how to weight cost-effectiveness estimates and severity of disease.

## Supplementary Material

Supplementary Data
