# Supplementary material for: Scaling-up essential neuropsychiatric services in Ethiopia: a cost-effectiveness analysis
Source: Health Policy Plan. 2015 Oct 21;31(4):504–13. doi: 10.1093/heapol/czv093 (PMC4986243; doi:10.1093/heapol/czv093)
Supplement: Supplementary Data [file supp_31_4_504__index.html]

Scaling-up essential neuropsychiatric services in Ethiopia: a cost-effectiveness analysis — Supplementary Data 

# Scaling-up essential neuropsychiatric services in Ethiopia: a cost-effectiveness analysis

## Supplementary Data

files

- Supplementary Data - pdf file
- Supplementary Data - pdf file
