# Supplementary material for: Scaling-up essential neuropsychiatric services in Ethiopia: a cost-effectiveness analysis
Source: Health Policy Plan. 2015 Oct 21;31(4):504–13. doi: 10.1093/heapol/czv093 (PMC4986243; doi:10.1093/heapol/czv093)
Supplement: Supplementary Data [file supp_czv093_TableS2.pdf]

Table S2

		Patient-related unit costs (in million US$)						Administrative costs (in million US$)		Total costs	
Disease Category	Intervention number	Inpatient care	Outpatient care	Primary Care	Psychosocial care	Drug costs	Labratory costs	Program costs	Traning costs	In million US$	per capita
Depression	DEP1	0.78	0.54	3.36	0.00	1.33	4.86	0.20	0.35	11.44	0.13
	DEP2	0.78	0.54	3.34	0.00	3.24	4.84	0.20	0.35	13.31	0.15
	DEP3	0.78	0.54	3.34	38.42	0.00	4.84	0.20	0.77	48.90	0.56
	DEP4	0.77	0.53	3.31	38.03	1.14	4.79	0.20	1.18	49.97	0.58
	DEP5	0.77	0.53	3.29	37.74	2.78	4.76	0.20	1.18	51.24	0.59
	DEP6	0.29	0.21	3.04	33.28	2.69	3.46	0.43	1.15	44.55	0.51
	DEP7	0.29	0.21	3.03	33.11	5.55	3.44	0.43	1.15	47.21	0.54
Schizophrenia	SCZ1	12.91	0.51	0.66	0.00	1.46	0.42	0.39	0.49	16.83	0.19
	SCZ2	12.05	0.51	0.66	0.00	1.48	1.26	0.39	0.49	16.83	0.19
	SCZ3	11.39	0.51	0.66	1.94	1.46	0.42	0.39	0.98	17.75	0.20
	SCZ4	11.39	0.51	0.66	1.94	1.48	1.26	0.39	0.98	18.60	0.21
	SCZ5	11.06	0.51	0.66	4.72	1.46	0.42	0.93	1.47	21.22	0.24
	SCZ6	11.06	0.51	0.66	4.72	1.48	1.26	0.93	1.47	22.07	0.25
Bipolar disorder	BIP1	0.83	0.65	0.28	0.00	13.00	4.83	0.29	0.62	20.50	0.24
	BIP2	0.77	0.65	0.28	0.00	18.71	1.58	0.29	0.62	22.90	0.26
	BIP3	0.79	0.65	0.28	1.79	13.01	4.83	0.29	2.07	23.71	0.27
	BIP4	0.73	0.65	0.28	1.79	18.73	1.58	0.29	2.07	26.14	0.30
Epilepsy	EPI1	0.40	1.02	4.67	0.00	1.64	12.95	0.29	0.93	21.90	0.25
	EPI2	0.40	1.02	2.33	0.00	17.80	25.18	0.29	0.93	47.95	0.55
