# Supplementary material for: Scaling-up essential neuropsychiatric services in Ethiopia: a cost-effectiveness analysis
Source: Health Policy Plan. 2015 Oct 21;31(4):504–13. doi: 10.1093/heapol/czv093 (PMC4986243; doi:10.1093/heapol/czv093)
Supplement: Supplementary Data [file supp_czv093_Appendix.pdf]

Table S1

Treatment	Drug/ other treamtent*	Patient adherence	Provider adherence	% at inpatient care (average number of days)	% at residential care (average number of days)	% at outpatient care (average number of visits)	% in primary care (average number of visits)	Psychotherapy (average number of session of 30 min)	% of case-managment**	Labratory tests	EEG
**Depression**											
Treatment with older Antidepressants (TCA)	Amtriptyline (100 mg daily)	47 %	70 %	5,8 % (9,8 days)		22 % (5,4 visits)	100% (5,8 visits)	0 %	0 %	83% (TSH, FT4, creatinin, glucose, Hb, ALAT)	
Treatment with newer Antidepressants (SSRI)	Fluoxetine(20 mg daily)	50 %	80 %	5,8 % (9,8 days)		22 % (5,4 visits)	100% (5,8 visits)	0 %	0 %	83% (TSH, FT4, creatinin, glucose, Hb, ALAT)	
Psychotherapy	Cognitive therapy (16-20 hours)	50 %	80 %	5,8 % (9,8 days)		22 % (5,4 visits)	100% (5,8 visits)	90 %(16,4 sessions)	0 %	83% (TSH, FT4, creatinin, glucose, Hb, ALAT)	
Comibnation of AD and psychotherapy	Amtriptyline / Fluoxetine, cognitive therapy	50%/53%	80%/90%	5,8 % (9,8 days)		22 % (5,4 visits)	100% (5,8 visits)	90 %(16,4 sessions)	0 %	83% (TSH, FT4, creatinin, glucose, Hb, ALAT)	
Proactive care	Amtriptyline / Fluoxetine, cognitive therapy	53%/53%	85%/90%	3,6 %(6,3 days)		13,5 % (3,6 visits)	100 % (4,40 visits	52,5 % (10,8 sessions)	90 % (4,8 visits)	83% (TSH, FT4, creatinin, glucose, Hb, ALAT)	
**Schizophrenia**											
Older antipsychotics	Chlorpromazine 300 mg daily/Fluphenazine deconate 25 mg daily	47 %	80 %	50% (35,3 days)	5,5 % (84 days)	50 % (4,5 visits)	50 % (4,2 visits)	0 %	0 %	100% (2 tests of fasting glucose, weigth) 23% (CBC package)	
Newer antipsychotics	Risperidone 4 mg daily	53 %	80 %	50% (32,4 days)	5,5 % (84 days)	50 % (4,5 visits)	50 % (4,2 visits)	0 %	0 %	100% (2 tests of fasting glucose, weigth, CBC package)	
Older antipsychotics + psychosocial treatment	Chlorpromazine 100 mg daily/Fluphenazine deconate 25 mg daily, supportive psychosocial treatment	50 %	80 %	46,5% (32,4 days)	5,5 % (84 days)	50 % (4,5 visits)	50 % (4,2 visits)	77 % (7,2 sessions of 30 min)	0 %	100% (2 tests of fasting glucose, weigth) 23% (CBC package)	
Newer antipsychotics + psychosocial treatment	Risperidone 4mg daily, supportive psychosocial treatment	57 %	80 %	46,5% (32,4 days)	5,5 % (84 days)	50 % (4,5 visits)	50 % (4,2 visits)	77 % (7,2 sessions of 30 min)	0 %	100% (2 tests of fasting glucose, weigth, CBC package)	
Case-management with older antipsychotics	Chlorpromazine 300 mg daily/Fluphenazine deconate 25 mg daily	60 %	80 %	44,8 % (32,4 days)	5,5 % (84 days)	50 % (4,5 visits)	50 % (4,2 visits)	77 % (7,2 sessions of 30 min)	58,8 % (9,0 visits)	100% (2 tests of fasting glucose, weigth) 23% (CBC package)	
Case-management with newer antipsychotics	Risperidone 4 mg daily	60 %	80 %	44,8 % (32,4 days)	5,5 % (84 days)	50 % (4,5 visits)	50 % (4,2 visits)	77 % (7,2 sessions of 30 min)	58,8 % (9,0 visits)	100% (2 tests of fasting glucose, weigth, CBC package)	
**Bipolar Disorder**											
Older Mood Stabilizer	Lithium Carbonate	43 %	80 %	12,5% (7,0 days)	3,1% (28,1 days)	100% (3,5 visits)	50 % (2,3 visits)	0 %		100% (4 tests p-Lithium, 1 test; TSH, FT4, creatinin and electrolytes)	
Newer Mood Stabilizer	Sodium Valproate	48 %	80 %	12,5% (6,0 days)	3,1% (28,1 days)	100% (3,5 visits)	50 % (2,3 visits)	0 %		100% (creatinin, ALAT, ASAT, CBC with plt) 50% (TSH, FT4)	
Older Mood Stabilizer + psychosocial	Lithium Carbonate + supportive psychososial treatment	48 %	80 %	11,3% (7,0 days)	3,1% (28,1 days)	100% (3,5 visits)	50 % (2,3 visits)	100 % (6,5 sessions of 30 minutes)		100% (4 tests p-Lithium, 1 test; TSH, FT4, creatinin and electrolytes)	
Newer Mood Stabilizer + psychosocial	Sodium Valproate + supportive psychosocial treatment	52 %	80 %	11,3 % (6,0 days)	3,1% (28,1 days)	100% (3,5 visits)	50 % (2,3 visits)	100 % (6,5 sessions of 30 minutes)		100% (creatinin, ALAT, ASAT, CBC with plt) 50% (TSH, FT4)	
**Epilepsy**											
Older antiepileptic	Phenobarbital 100 mg daily	70 %	90 %	3% (5 days)		25% (4 visits)	100 % (2 days)			100% (Liver tests***, CBC package	50 %
Newer antiepileptic	Carbamazepine 600 mg daily	70 %	90 %	3% (5 days)		25% (4 visits)	100 % (2 days)			100% (CBC package, plt, s-I,Liver test***, BUN, Urinanalysis, thyroid tests, s-Na)	50 %

*Active treatment with antidepressants and psychotherapy is given to pateints with moderate to severe depression. Mild depression is handled in primary care. Evidence show no effect of medical treatment for mild depression.

** Depression: manitainance treatment and follow-up to prevent relapse. Schizophrenia: case-finding of patients and extra follow-up.

*** Liver tests include: bil-tot, bil-dir, ASAT, ALAT and ALP
